# Prognostic Significance of Triglyceride Glucose Index in Intracerebral Hemorrhage

**DOI:** 10.3390/medsci14020172

**Published:** 2026-03-31

**Authors:** Giovanni Baronchelli, Francesco Berinato, Maddalena Toffali, Giacomo Urbinati, Stefano Forlivesi, Mario Sebastiani, Chiara Tolassi, Irene Girotto, Giorgio Busto, Enrico Fainardi, Ilaria Casetta, Michele Laudisi, Andrea Zini, Andrea Pilotto, Andrea Morotti, Alessandro Padovani

**Affiliations:** 1 Neurology Unit, Department of Clinical and Experimental Sciences, University of Brescia, 25123 Brescia, Italy; g.baronchelli007@unibs.it (G.B.);; 2 Neurology Unit, Department of Continuity of Care and Frailty, ASST-Spedali Civili, 25123 Brescia, Italy; 3 IRCCS Istituto Delle Scienze Neurologiche Di Bologna, Department of Neurology and Stroke Center, Maggiore Hospital, 40100 Bologna, Italy; 4 Neurobiorepository and Laboratory of Advanced Biological Markers, University of Brescia and ASST Spedali Civili Hospital, 25123 Brescia, Italy; 5 Department of Biomedical Experimental and Clinical Science, Neuroradiology, University of Firenze, AOU Careggi, 50134 Firenze, Italy; 6 IRCCS San Camillo Hospital, 30126 Venice, Italy; 7 Department of Neurosciences and Rehabilitation, Azienda Ospedaliero-Universitaria di Ferrara Arcispedale Sant’Anna-Cona, Ferrara University of Ferrara, 44124 Ferrara, Italy; 8 Department of Neurobiology, Care Sciences and Society (NVS) Center for Alzheimer Research, Division of Clinical Geriatrics, Karolinska Institutet, 17177 Stokolhm, Sweden; 9 Brain Health Center, University of Brescia, 25123 Brescia, Italy

**Keywords:** intracerebral hemorrhage, triglycerides, TyG-i, functional outcome

## Abstract

**Background**: The triglyceride glucose index (TyG-i), a biomarker of insulin resistance, has been associated with adverse vascular outcomes and risk stratification in several cardiovascular and cerebrovascular phenotypes. However, data on TyG-i as a prognostic marker in spontaneous intracerebral hemorrhage (ICH) remain limited. **Objective:** To explore the association between TyG-i and 90-day functional outcome in patients with ICH. **Methods**: A retrospective analysis of adult patients admitted for non-traumatic small vessel disease-related ICH at three Italian neurological institutions was conducted. TyG-i was calculated on admission as Ln[(fast triglycerides (mg/dL) × fast glucose (mg/dL))]/2. Functional outcome was measured with the modified Rankin Scale (mRS) at 90 days from the index event. TyG-i was analyzed as a continuous variable and categorized in quintiles (Q1 to Q5). Predictors of poor outcome (mRS 4–6) were investigated with multivariable logistic regression. **Results**: A total of 463 patients were included, of whom 197 (42.5%) had poor outcome at 90 days. TyG-i analyzed as a continuous variable was not associated with unfavorable prognosis. TyG-i analyzed as a categorical variable stratified by quintiles showed a non-linear U-shaped relationship with functional outcome; patients in Q4 had the lowest risk of poor outcome (Q1 reference, OR 0.44, 95% CI 0.22–0.87, *p* = 0.019). **Discussion**: We found a potential non-linear relationship between TyG-i and ICH outcome, with higher odds of good prognosis in patients with intermediate values. **Conclusions**: TyG-i may represent a promising, low-cost and widely available biomarker that might improve prognostication in clinical practice, but further studies are needed.

## 1. Introduction

Spontaneous (non-traumatic) intracerebral hemorrhage (ICH) is a severe stroke subtype with high early mortality and long-term disability, and early prognostication remains clinically important for triage, counseling, and trial design [1].

Established predictors of functional outcome include age, level of consciousness (e.g., Glasgow Coma Scale, GCS), hematoma volume and location, and intraventricular extension; these elements are incorporated into widely used severity scales (e.g., the ICH Score) [2]. Nevertheless, ICH remains a heterogeneous entity, and additional, easily obtainable biomarkers capturing underlying vascular vulnerability and systemic pathophysiology may refine prognostic stratification—particularly when they are mechanistically linked to the biological basis of vessel rupture and secondary brain injury pathways [1].

From a pathophysiological perspective, most “primary” spontaneous ICH arises from rupture of small penetrating arteries damaged by cerebral small vessel disease (cSVD), classically hypertensive arteriolosclerosis and cerebral amyloid angiopathy (CAA). Beyond the immediate mass effect and intracranial pressure rise (primary injury), hematoma expansion, perihematomal edema, blood–brain barrier (BBB) disruption, oxidative stress, and neuroinflammation drive secondary injury and influence functional recovery [1]. Contemporary conceptual models increasingly frame ICH as an acute manifestation of underlying cSVD, integrating ICH with subclinical markers such as cerebral microbleeds (CMBs) and white matter hyperintensities (WMHs) [3].

Insulin resistance (IR) is increasingly recognized as a systemic vascular stressor that can plausibly contribute to cSVD and hemorrhage-prone microangiopathy through convergent mechanisms: endothelial dysfunction (including impaired nitric oxide signaling), chronic low-grade inflammation, oxidative stress, vascular remodeling, and BBB impairment. Recent mechanistic and clinical reviews summarize accumulating evidence that IR is associated with cSVD imaging markers such as lacunes, enlarged perivascular spaces, and CMB, suggesting that disordered glucose–lipid metabolism may be linked to small vessel injury in the brain [4]. In support of this, observational data indicate that IR is associated with the presence of CMBs, an imaging correlate of hemorrhage-prone microangiopathy and a marker of increased risk for future symptomatic ICH [5].

Several lines of evidence connect TyG-i to intermediate vascular phenotypes that plausibly bridge IR and small-vessel rupture. First, TyG-i has been associated with arterial stiffness and with subclinical atherosclerosis in meta-analyses and large cohort studies [6]. Arterial stiffness can promote microvascular damage and cSVD progression [7]. Second, TyG-i has been directly linked to overall cSVD burden and related outcomes in clinical cohorts, reinforcing its potential relevance to the microangiopathic substrate that underlies most primary ICH [8].

The triglyceride glucose index (TyG-i) is a simple composite derived from fasting-intended triglycerides and fasting-intended glucose; it was originally proposed as a surrogate marker of IR and has shown meaningful correlation with the hyperinsulinemic–euglycemic clamp, while remaining feasible for large-scale use because it relies on routinely available laboratory values [9,10]. TyG-i has been associated with incident cardiovascular events and stroke in multiple cohorts, supporting its role as an integrative marker of metabolic and vascular risk [11].

A complementary rationale for studying TyG-i in ICH is that early metabolic dysregulation influences acute ICH evolution and outcomes. Stress-related hyperglycemia is common after ICH and, when quantified as a stress–hyperglycemia ratio (SHR; admission glucose adjusted by chronic glycemic status using hemoglobin A1c, HbA1c), has been associated with hematoma expansion and poor outcomes in spontaneous ICH [12]. Because TyG-i integrates glucose with triglycerides (a metabolic substrate influenced by IR and systemic stress), it may capture a biologically relevant metabolic milieu at presentation plausibly affecting secondary injury and recovery trajectories [13].

In contrast, the prognostic value of TyG-i in ICH remains largely under-investigated. To date, available evidence on TyG-i in the context of intracerebral hemorrhage is limited and mainly derived from critically-ill populations, with a primary focus on short-term mortality rather than functional recovery [14,15]. Moreover, prior studies did not explore potential non-linear associations between TyG-i and outcome, nor did they specifically evaluate patients with spontaneous small vessel disease-related ICH.

Therefore, we aimed to explore the association between TyG-i and 90-day functional outcome (mRS) in patients with spontaneous ICH.

## 2. Materials and Methods

### 2.1. Standard Protocol Approvals, Registrations, and Patient Consents

All the study procedures were approved by relevant authorities (institutional review board) at each participating site (Arcispedale S. Anna, Ferrara, Italy (PN 26032009-15122011, date of approval: 15 December 2011); ASST Spedali Civili, Brescia, Italy (PN 4067-08052020, date of approval: 8 May 2020); IRCCS Istituto delle Scienze Neurologiche, Bologna, Italy (DL 196/2003, date of approval: 2015)). Written informed consent was obtained from patients, relatives, or legal representatives or waived by the institutional review board.

### 2.2. Study Population

A retrospective analysis of adult patients admitted for non-traumatic small vessel disease-related ICH at three Italian neurological institutions was conducted.

**Figure 1:**
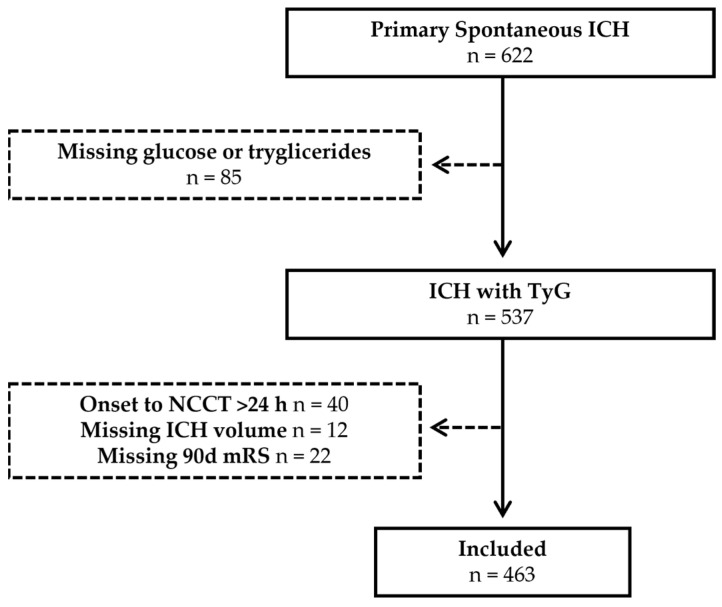
Study selection flowchart. Solid arrows indicate the included patients; dashed arrows indicate the excluded patients.

The main inclusion criteria were as follows: (1) age ≥ 18, (2) admission within 24 h from symptom onset (in patients with unclear symptom onset < 24 h from the time last seen well), (3) availability of baseline imaging and (4) availability of baseline routine blood tests performed on admission. The study selection flowchart is illustrated in Figure 1.

We excluded patients with any of the following: (1) traumatic brain injury, (2) ICH secondary to macrovascular lesion, (3) hemorrhagic conversion of ischemic stroke, (4) ICH secondary to any other brain disorder, (4) missing baseline non-contrast CT imaging, (5) missing functional outcome at 90 days and (6) patients undergoing surgical treatment for any indication (hematoma evacuation, external ventricular derivation or other surgical treatment).

### 2.3. Clinical Variables

The following clinical data were collected by trained investigators through interviews with patients and family members and review of medical charts: age, sex, history of diabetes, hypertension and dyslipidemia, antithrombotic therapy (antiplatelet and anticoagulant therapy) and statin treatment. The baseline clinical status was assessed with the Glasgow Coma Scale (GCS) and National Institute of Health Stroke Scale (NIHSS) on admission. Systolic blood pressure, fasting glucose and triglycerides, total cholesterol, and low- and high-density lipoprotein (LDL and HDL) levels were collected after hospital admission. Functional outcome was evaluated using the modified Rankin Scale (mRS) at 90 days from the index event. Follow-up outcome was obtained from outpatient service evaluation, review of medical charts or querying the national social security system for mortality data when available. All the mRS raters were blinded to the remaining clinical and imaging data.

Medical management followed the American Stroke Association and European Stroke Association Guidelines [1,16].

### 2.4. TyG-i Calculation

The TyG-i was calculated according to the following formula: Ln [fast triglycerides (mg/dL) × fast glucose (mg/dL)]/2 [9].

### 2.5. Imaging Variables

All patients underwent a baseline non-contrast computed tomography (NCCT) scan within 24 h from symptom onset or time last known well. The imaging acquisition parameters were not standardized and was based on local imaging protocols for NCCT acquisition in patients with acute stroke.

Follow-up NNCT scan was performed between 24 and 72 h after the onset, or earlier in case of clinical deterioration. The presence of intraventricular hemorrhage (IVH) was evaluated, and ICH location was classified as supratentorial or infratentorial. Among supratentorial ICH, we distinguished deep (thalamus or basal ganglia) and lobar ICH location. ICH volumes were calculated with a semiautomated, computer-assisted planimetric software (ITK-SNAP 4.0.2 or Stroke VCAR AW SERVER 3.2 est 4.6, GE HealthCare, Chicago, IL, USA). All the imaging raters were blinded to the main outcomes of interest of the study. We tested for inter-rater reliability across three imaging raters (AM, GB, EF) from two different centers and observed good reliability for ICH volume quantification, ICH location and presence of IVH (Cohen’s K and ICC > 0.80).

### 2.6. Statistical Analysis

Continuous variables were expressed as median or mean based on their parametric or non-parametric distribution (evaluated with the Shapiro–Wilk test) and compared with Mann–Whitney, T test or ANOVA as appropriate. Categorical variables were summarized as *n* (%) and compared with Chi-Square test. Spearman’s Rho was used for correlations between continuous variables. Poor functional outcome at 90 days, defined as mRS 4–6, was the main outcome of interest and its predictors were explored with binary logistic regression, adjusted for known predictors of death and poor functional outcome in ICH such as age, baseline ICH volume, admission GCS, infratentorial location and IVH presence. Every subject’s predicted probability of poor outcome was then calculated from individual data and logistic regression estimates and expressed as a continuous variable (range 0 to 1). TyG-i was analyzed as a continuous variable and stratified by quintiles to explore non-linear associations. Several secondary analyses were performed. First, the logistic regression accounted also for diabetes and statin treatment. Second, all potential confounders with *p* < 0.1 in univariate analysis were included in the logistic regression models as well. Third, we adjusted the logistic regression analyses also for follow-up ICH volume, to account for the occurrence of ICH expansion [17]. Fourth, we performed a sensitivity analysis excluding patients with diabetes. Finally, poor prognosis was defined as mRS 3–6 at 90 days from the index event. All the analyses were performed with SPSS V 25.0 and statistical significance was set at *p* < 0.05.

## 3. Results

A total of 463 subjects were included (median age 73 years, interquartile range (IQR) 66–81, 45.8% males), of whom 197 (42.5%) had poor outcome at 90 days. The study cohort characteristics are summarized in Table 1.

Table 2 illustrates the comparison between patients with good (mRS 0–3) versus poor (mRS 4–6) functional outcome at 90 days.

Patients with poor outcome had lower admission TyG-i (median 4.67, IQR 4.54–4.87 vs. 4.73, IQR 4.59–4.87, *p* = 0.031) and TyG-i did not correlate with measures of ICH severity, such as admission GCS (Rho −0.010, *p* = 0.835) and baseline ICH volume (Rho 0.053, *p* = 0.258). The inverse association between TyG-i and functional outcome observed in univariate analysis was not confirmed when accounting for potential confounders in multivariable binary logistic regression (odds ratio (OR) per one point increase in TyG-i, 0.59, 95% confidence interval (CI) 0.26–1.37, *p* = 0.220). However, when TyG-i was analyzed as a categorical variable stratified by quintiles, we observed a U-shaped relationship between TyG-i and functional outcome, as summarized in Table 3, with patients in Q4 having the lowest risk of unfavorable prognosis (OR 0.44, 95% CI 0.22–0.87, *p* = 0.019).

**Figure 2:**
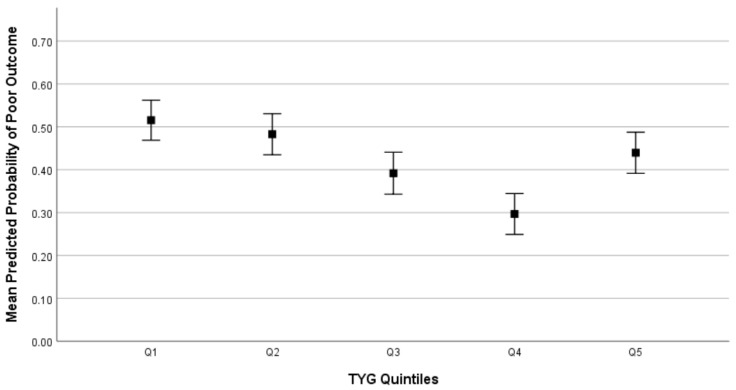
Predicted probability of poor outcome. Triglyceride and glucose index (TyG-i) quintiles: Q1 < 4.54, Q2 4.55–4.65, Q3 4.66–4.76, Q4 4.77–4.92, Q5 > 4.93. Mean predicted probability of poor outcome: Q1 52%, Q2 48%, Q3 39% Q4 31%, Q5 44%, *p* < 0.001.

These findings were confirmed in secondary analyses accounting also for diabetes, statin treatment and other variables with *p* < 0.1 in univariate analysis. We also tested for the presence of a linear trend across TyG-i quintiles and found no statistical significance (*p* = 0.106). All the main findings were also consistent after exclusion of patients with a history of diabetes. Figure 2 shows the predicted probability of poor outcome by TyG-i quintiles. The same U-shaped relationship between TyG-i quintiles and outcome was confirmed when unfavorable outcome was defined as mRS 3–6 (mean predicted probability of poor outcome: Q1 68%, Q2 67%, Q3 61%, Q4 54%, Q5 59%, *p* < 0.001).

## 4. Discussion

The main result of our analysis was that a quintile-based approach suggested a non-linear pattern between admission TyG-i and 90-day functional outcome after ICH. Specifically, patients in Q4 showed the lowest adjusted odds of poor outcome compared with Q1, whereas the remaining quintiles (Q2, Q3, and Q5) were not significantly different from the reference group. These findings are best interpreted as exploratory and hypothesis-generating. TyG-i analyzed as a continuous variable was not independently associated with functional outcome, and formal testing for non-linearity with flexible statistical approaches (e.g., restricted cubic splines) was not performed. While the quintile-based pattern is consistent with a non-linear relationship, only Q4 reached statistical significance relative to Q1, and no statistically significant linear trend was detected across quintiles (*p* = 0.106). The selective significance of Q4 rather than a broader middle range may reflect the limited statistical power of quintile-based stratification rather than a precise threshold effect. The observed pattern should therefore be regarded as a signal warranting further investigation, and any claims regarding the clinical applicability of TyG-i in ICH prognostication remain premature pending prospective validation.

The biological interpretation of our results remains uncertain because TyG-i is a composite index integrating triglycerides and glucose, both of which may reflect chronic metabolic status as well as acute stress physiology. Higher TyG-i values are commonly interpreted as a proxy of insulin resistance and may identify patients with a greater burden of vasculometabolic risk [18]. Conversely, lower TyG-i values may reflect frailty, poor nutritional status, or systemic catabolism [19], factors that have been associated with worse outcomes after ICH [20]. Poor nutritional status might in turn be associated with a higher risk of infections, a common determinant of neurological deterioration and an independent prognostic predictor in patients with ICH [20,21]. It must be acknowledged, however, that our study does not directly measure nutritional status, frailty, inflammatory markers, or in-hospital complications such as infections; the proposed mechanistic interpretations therefore remain speculative and are not directly supported by biological evidence from our dataset.

These findings could be in line with emerging evidence of U-shaped associations between TyG-i and mortality or cardiovascular outcomes in both adult and pediatric populations [22,23]. Previous studies on the significance of TyG-i in patients with ICH observed a direct relationship between this biomarker and several post-operative outcomes including mortality [14,15]. Several factors might account for this partial discrepancy with our findings, such as different settings (intensive care unit versus stroke unit and general neurology), different populations (patients undergoing surgery versus patients managed with medical therapy only), lack of adjustment for different TyG-i strata (therefore missing non-linear associations) and use of mortality as the only outcome of interest in previous reports.

TyG-i might also act as a non-specific acute-phase reactant, with a proportional increase that correlates with ICH severity. Our data does not support this hypothesis, as we did not observe a significant association between TyG-i and the main known determinants of ICH prognosis [24]. In particular, we did not observe a significant association between this biomarker and ICH volume. This, however, does not exclude an acute-phase component, particularly because fasting status and sampling timing were not standardized and chronic glycemic history (HbA1c) was unavailable. In this regard, stress-related hyperglycemia metrics accounting for chronic glycemic exposure (e.g., stress–hyperglycemia ratio) have been associated with hematoma expansion and poor outcome after spontaneous ICH, highlighting the potential importance of timing and stress physiology when interpreting early metabolic biomarkers. Therefore, our findings should not be interpreted as evidence that TyG-i is independent of acute metabolic responses in ICH.

From a clinical point of view, accurate ICH prognostication remains an unmet need and TyG-i might improve the identification of patients at risk of poor outcome. The discriminative performance of prognostic scores remains suboptimal, suggesting the opportunity to integrate these tools with other biomarkers of outcome in ICH [25]. Importantly, our study did not formally assess the incremental predictive value of TyG-i over established prognostic models (e.g., through comparison of C-statistics, AUC, net reclassification index, or integrated discrimination improvement). Any claims regarding the clinical utility of TyG-i for ICH prognostication should therefore be regarded as premature and require formal model comparison in prospective studies.

Furthermore, if the association between insulin resistance and ICH outcomes were confirmed in prospective studies, insulin resistance could represent an interesting target for future interventional research, although no causal inference can be drawn from the present observational data.

Current prognostic algorithms are mainly based on clinical and imaging variables such as age, clinical severity, ICH size and location and presence of intraventricular extension [26,27]. It therefore seems plausible that a blood-based biomarker, capturing other biological mechanisms of ICH natural history, might provide an added value and improve prognostication. TyG-i levels should not be used alone to guide prognostication and full medical support is recommended for all ICH patients in the first 24–48 h from admission, as consistent with guideline recommendations [1].

Our findings are best interpreted as hypothesis generating, suggesting the need for further research to prospectively confirm our observations and provide a better characterization of the underlying biology. It might also be of interest to explore whether TyG-i is associated with other biological markers of inflammation and insulin resistance.

Some limitations should be acknowledged. First, our findings were derived from a retrospective analysis, raising the possibility of selection bias and confounding by unmeasured variables. Second, we had limited data on potential confounders, such as nutritional status and medical complications that might impact ICH prognosis, such as infections [21]. Third, BMI was not available in all the included subjects and might account for some of our findings. However, this possibility appears unlikely, as our data showed similar BMI and obesity rates between patients with good and poor outcome. Furthermore, the previous literature suggests that overweight or obese patients with ICH did not have worse outcomes [28]. Fourth, the ICH recovery trajectory is long, with improvement up to one year from the index event, and only the 90-day outcome was available in our dataset [29]. Fifth, it remains to be determined whether the timing of TyG-i analysis might influence its association with key prognostic variables and its added value for accurate prognostication. Furthermore, TyG-i relies on fasting-intended triglycerides and glucose. In the acute ICH setting, strict fasting is challenging. Across centers, lipid/glucose sampling was obtained early during hospitalization (within 24 h after admission) as part of routine care according to standard clinical practice, but fasting duration, exact sampling time relative to last caloric intake, and early medical interventions were not consistently documented in a harmonized manner. This variability represents a potential source of measurement bias. Sixth, we were unable to account for early withdrawal or limitation of care, a critical and known predictor of outcome leading to self-fulfilling prophecies [30]. Seventh, TyG-i was evaluated at a single time point after ICH. It might be valuable to explore its longitudinal changes and determine the sampling time with the highest added value for prognostication in patients with acute ICH. Eight, the exclusion of patients undergoing surgical treatment was primarily due to the fact that these individuals were managed within the Neurosurgery Unit, and therefore their clinical and imaging data were not accessible to our research team. As a result, we were unable to reliably include these cases in our database and subsequent analyses. This approach increased cohort homogeneity by focusing on medically managed small vessel disease-related ICH, but limits generalizability to more severe or surgically treated cases. We acknowledge that this methodological choice may limit the generalizability of our findings and introduce potential selection bias, including a collider bias effect, by preferentially enriching the study cohort with less-severe cases. Finally, it might be of interest to explore ICH etiology-related differences in the prognostic role of TyG-I and to develop more complex and comprehensive statistical approaches to test for non-linear associations (i.e., restricted cubic splines).

## 5. Conclusions

TyG-i may represent a promising biomarker associated with ICH outcome and patients with intermediate levels seem to have the highest odds of favorable functional outcome. Our findings are best interpreted as hypothesis generating, warranting further studies to prospectively validate our observations in larger cohorts and provide a better characterization of the underlying biological mechanisms.

## Figures and Tables

**Table 1:** Cohort characteristics.

	ALL
	*N* = 463
Age, median (IQR)	73 (66–81)
Sex, males, *n* (%)	212 (45.8)
Hypertension, *n* (%)	341 (73.7)
Diabetes, *n* (%)	72 (15.6)
Dyslipidemia, *n* (%)	144 (31.1)
Antiplatelets, *n* (%)	141 (30.5)
Anticoagulation, *n* (%)	54 (11.7)
Statin, *n* (%)	67 (14.5)
Baseline volume, median (IQR), mL	12 (5–29)
Baseline NCCT timing, median, (IQR), h	4 (3–10)
Follow-up volume, median (IQR), mL	14 (6–35)
GCS, median (IQR)	15 (13–15)
SBP, median (IQR), mmHg	155 (140–180)
IVH presence, *n* (%)	114 (24.6)
Infratentorial location, *n* (%)	26 (5.6)
Total cholesterol, median (IQR), mg/dL	188 (161–220)
HDL cholesterol, median (IQR), mg/dL	46 (27–57)
LDL cholesterol, median (IQR), mg/dL	120 (−97–143)
Triglycerides, median (IQR), mg/dL	100 (75–137)
Glucose, median (IQR), mg/dL	119 (101–146)
BMI, median (IQR), mg/dL *	25.0 (22.8–27.8)
BMI > 30, *n* (%) *	41/219 (18.7)
TyG-i, median (IQR)	4.71 (4.56–4.87)

* Available in 219 subjects.

**Table 2:** Study population stratified by functional outcome.

	Good Outcome	Poor Outcome	*p*
	*N* = 266	*N* = 197	
Age, median (IQR)	71 (61–77)	78 (68–83)	<0.001
Sex, males, *n* (%)	150 (56.4)	101 (51.3)	0.274
Hypertension, *n* (%)	180 (67.7)	161 (81.7)	<0.001
Diabetes, *n* (%)	43 (16.2)	29 (14.7)	0.672
Dyslipidemia, *n* (%)	75 (28.2)	69 (35.0)	0.116
Antiplatelets, *n* (%)	78 (29.3)	63 (32.0)	0.539
Anticoagulation, *n* (%)	17 (6.4)	37 (18.8)	<0.001
Statin, *n* (%)	30 (11.3)	37 (18.8)	0.023
Baseline volume, median (IQR), mL	10 (4–17)	20 (8–43)	<0.001
Baseline NCCT timing, median, (IQR), h	4 (3–9)	4 (3–11)	0.747
Follow-up volume, median (IQR), mL	10 (5–21)	24 (9–55)	<0.001
GCS, median (IQR)	15 (13–15)	14 (11–15)	0.011
SBP, median (IQR), mmHg	150 (140–180)	160 (140–190)	0.106
IVH presence, *n* (%)	41 (15.4)	73 (37.1)	<0.001
Infratentorial location, *n* (%)	13 (4.9)	13 (6.6)	0.429
Total cholesterol, median (IQR), mg/dL	198 (163–224)	182 (154–215)	0.019
HDL cholesterol, median (IQR), mg/dL	46 (35–57)	45 (38–56)	0.645
LDL cholesterol, median (IQR), mg/dL	123 (98–145)	115 (94–141)	0.095
Triglycerides, median (IQR), mg/dL	108 (84–145)	92 (72–123)	<0.001
Glucose, median (IQR), mg/dL	112 (100–143)	123 (105–152)	0.005
BMI, median (IQR), mg/dL *	25.4 (23.6–28.8)	24.7 (22.0–27.3)	0.192
BMI > 30, *n* (%) *	22/98 (22.4)	19/121 (15.7)	0.203
TyG-i, median (IQR)	4.73 (4.59–4.87)	4.67 (4.54–4.87)	0.031

Interquartile range (IQR), non-contrast computed tomography (NCCT), Glasgow Coma Scale (GCS), National Institute of Health Stroke Scale (NIHSS), systolic blood pressure (SBP), intraventricular hemorrhage (IVH), low-density lipoprotein (LDL), high-density lipoprotein (HDL), triglyceride and glucose index (TyG-i). * Available in 219 subjects.

**Table 3:** Multivariable association between TyG-i and poor functional outcome.

TyG-i Quintiles	OR (95% CI)	*p*
Q1 *n* = 97	Reference	NA
Q2 *n* = 87	0.79 (0.41–1.53)	0.483
Q3 *n* = 97	0.59 (0.31–1.13)	0.109
Q4 *n* = 91	0.44 (0.22–0.87)	0.019
Q5 *n* = 91	0.71 (37–1.36)	0.298

Triglyceride and glucose index (TyG-i), odds ratio (OR), confidence interval (CI), not available (NA). Poor functional outcome defined as modified Rankin Scale 4–6 at 90 days. Logistic regression adjusted for age, baseline ICH volume, admission GCS, infratentorial location and IVH presence. TyG-i quintiles: Q1 < 4.54, Q2 4.55–4.65, Q3 4.66–4.76, Q4 4.77–4.92, Q5 > 4.93.

## Data Availability

Requests to access the dataset might be sent to the corresponding author due to privacy and ethical restrictions.

## Abbreviations

The following abbreviations are used in this manuscript:

BBB	Blood–brain barrier
BMI	Body mass index
CAA	Cerebral amyloid angiopathy
CMBs	Cerebral microbleeds
cSVD	Cerebral small vessel disease
CI	Confidence interval
GCS	Glasgow Coma Scale
HbA1c	Hemoglobin A1c
HDL	High-density lipoprotein
ICH	Intracerebral hemorrhage
IR	Insulin resistance
IQR	Interquartile range
IVH	Intraventricular hemorrhage
LDL	Low-density lipoprotein
mRS	Modified Rankin Scale
NCCT	Non-contrast computed tomography
NIHSS	National Institute of Health Stroke Scale
OR	Odds ratio
SBP	Systolic blood pressure
SHR	Stress–hyperglycemia ratio
TyG-i	Triglyceride glucose index
WMH	White matter hyperintensities
